# Transcriptomic Meta‐Analysis Reveals Hub Genes Integrating Multiple Abiotic Stress Responses in Wheat

**DOI:** 10.1002/fsn3.70909

**Published:** 2025-09-08

**Authors:** Minqiang Ding, Sajid Fiaz, Kui Wan, Haowen Chang, Rui Pan, Tayachew Admas

**Affiliations:** ^1^ College of Agriculture Yangtze University Jingzhou China; ^2^ Institute of Molecular Biology and Biotechnology The University of Lahore Lahore Pakistan; ^3^ Department of Biology, College of Natural and Computational Sciences Mizan‐Tepi University Tepi Ethiopia

**Keywords:** RNA‐seq, stress‐resilient crop, transcription factors, *Triticum aestivum*, WGCNA

## Abstract

Climatic challenges increasingly threaten global food security, necessitating crops with enhanced multi‐stress resilience. Through systematic transcriptomic analysis of 100 wheat genotypes under heat, drought, cold, and salt stress, we identified 3237 differentially expressed genes (DEGs) enriched in key stress‐response pathways. Core transcription factors (*MYB*, *bHLH*, *HSF*) and two functional modules governing abiotic tolerance were characterized. Phenotypic assessments revealed significant stress‐induced alterations in plant height, biomass, and chlorophyll content. RT‐qPCR confirmed marked upregulation of eight candidate genes, including *BES1*/*BZR1* and *GH14*, across most stresses, indicating their critical role in wheat's adaptive responses. This study aims to identify potential genes linked to multiple abiotic stress tolerance and provides an experimental foundation as well as theoretical support for the future development of stress‐resilient crops.

## Graphical abstract

This study focuses on wheat's response to multiple abiotic stresses, including heat, cold, drought, and salt stress. It exhibits phenotypic changes, including reductions in plant height, biomass, and chlorophyll content, in response to these stresses. A meta‐analysis of transcriptomes identified 3237 multiple abiotic resistance genes, of which eight hub genes were recognized. It lays a foundation for future breeding of widely stress‐resilient crops, aiming to enhance global food security amid climatic challenges.

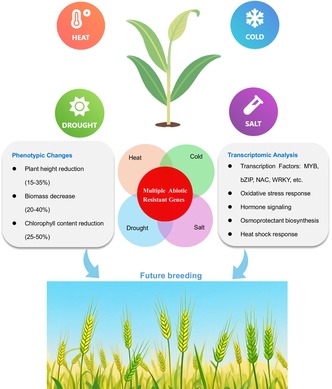

## 1 Introduction

Wheat ( *Triticum aestivum* L.) is a crucial global food crop, serving as a staple for approximately two‐thirds of the population while providing ~20% of daily dietary protein (Sandhu et al. 2022). However, abiotic stresses, including drought, heat, cold, and salinity, substantially reduce wheat yield (Abhinandan et al. 2018). The increased frequency of extreme weather events elevates the likelihood of plants being exposed to temperature extremes, salinity, drought, and heavy rainfall (Sun et al. 2021; Waqas et al. 2021; Zafar et al. 2020). Consequently, crops frequently encounter multiple stresses, underscoring the urgent need to identify genes conferring tolerance to combinatorial challenges.

Plants develop adaptive morphological and physiological responses to abiotic stresses (Nadeem et al. 2023; Priya et al. 2023; Zhang et al. 2022). Variability in concentrations of specific phytohormones, particularly those associated with stress responses, represents one of the critical adjustments made during exposure to abiotic stress. Stress‐related phytohormones are key to promoting plant development and aiding plants to cope with diverse biological and abiotic conditions, especially under drought and salinity‐induced stress (Cai et al. 2014; Ji et al. 2013; Yoshida et al. 2015). Abiotic stress further elevates reactive oxygen species (ROS) accumulation, disrupting cellular homeostasis (Kumar et al. 2017). Notably, multiple stresses often elicit convergent physiological responses, suggesting a shared genetic regulatory mechanism.

By adjusting physiological processes to sustain growth and development, plants can withstand a variety of abiotic stress factors, including drought, high and low temperatures, and salinity stress conditions. To date, research has primarily focused on plant responses to individual stressors (Dossa et al. 2017; Wang et al. 2016; Zhang, Li, et al. 2019). Although these studies are valuable for crop stress tolerance improvement, field‐grown wheat typically experiences multiple environmental constraints simultaneously during development, often causing greater yield losses than singular stresses (Jiang et al. 2025). Transcriptome analyses have identified numerous differentially expressed genes (DEGs) and highlighted transcription factors' (TFs) importance under stress. Recent studies demonstrate that biological pathways undergo extensive rewiring during stress adaptation, with gene expression profiling revealing complex regulatory networks under combinatorial stresses (Zhang, Ali, et al. 2019).

Despite these advances, large‐scale integrative transcriptomic analyses identifying core hub genes for multiple abiotic stress tolerance in wheat remain scarce. This represents a critical knowledge gap in functional genomics and stress‐resilient breeding (Nazari and Zinati 2023; Nemati et al. 2023; Saidi et al. 2023). Meta‐analysis provides a robust framework for integrating heterogeneous transcriptomic datasets, overcoming batch effects by emphasizing consistent expression trends across independent studies and reducing the influence of study‐specific results (Shokri‐Gharelo et al. 2024; Tahmasebi et al. 2019). Moreover, by aggregating effect sizes or differential expression patterns from multiple experiments, meta‐analysis increases statistical power and filters out spurious signals, thereby mitigating dataset variability (Rawat et al. 2015; Sircar and Parekh 2019; Tahmasebi et al. 2019). This approach helps to reveal genes that are reliably responsive to stress conditions across different experiments and platforms (Pan et al. 2022; Rawat et al. 2015). Although meta‐transcriptomic approaches have successfully identified stress‐responsive hub genes in crops such as rice for drought and heat tolerance (Derakhshani et al. 2024; Zhu et al. 2019), maize for salinity stress (Zinati and Nazari 2023), and soybean for cold and flooding responses (Kazemi et al. 2023), wheat presents distinct challenges that necessitate a dedicated integrative analysis. As a globally cultivated hexaploid crop with complex genome interactions, wheat exhibits stress response mechanisms that are less conserved relative to diploid species like rice and soybean (Barratt et al. 2023; Nazari and Zinati 2023; Shokri‐Gharelo et al. 2024). Moreover, wheat exceeds most major cereals in vulnerability to concurrent abiotic stresses because of its cultivation in marginal environments where drought, heat, and salinity frequently coincide (Azad et al. 2024; Saidi et al. 2023; Shokri‐Gharelo et al. 2024). Despite extensive public transcriptomic resources, analogous meta‐analyses remain critically underdeveloped for wheat (Rawat et al. 2015; Tahmasebi et al. 2019), impeding the development of climate‐resilient varieties needed to safeguard global food security.

To address this gap, we conducted a meta‐analysis of 100 RNA‐seq datasets encompassing drought, salinity, and temperature extremes in wheat. Using weighted gene co‐expression network analysis (WGCNA), we identified eight key DEGs with multi‐stress resistance potential. Gene Ontology (GO) and Kyoto Encyclopedia of Genes and Genomes (KEGG) pathway annotation revealed functional associations, followed by experimental validation of candidate genes via real‐time fluorescence quantitative PCR (RT‐qPCR) and overexpression assays. This integrative approach accelerates the discovery of breeding‐relevant tolerance genes.

## 2 Methods

### 2.1 RNA‐Seq Data Acquisition and Analysis

A total of 100 RNA‐seq datasets were systematically retrieved from 10 independent studies in the NCBI SRA database (accession codes provided in Table S1) using Aspera Connect v3.6.2. Dataset selection followed stringent criteria: (1) inclusion of both control and stress‐treated samples; (2) a minimum of three biological replicates per condition; (3) clear experimental documentation of stress application protocols; (4) Q30 score > 85% in quality assessment using FastQC v0.11.9. SRA files were converted to FASTQ format using fastq‐dump v2.8.0. Quality control was performed with fastp v0.20.1 (parameters: ‐‐adapter_sequence = auto, ‐‐qualified_quality_phred 20, ‐‐length_required 50). Reads were aligned to the IWGSC RefSeq v2.1 wheat reference genome using HISAT2 v2.2.1 (parameters: ‐‐dta ‐‐phred33 ‐‐max‐intronlen 5000). Resulting SAM files were converted to sorted BAM files using samtools v1.10 (‐q 20). Gene expression quantification was performed via featureCounts v2.0.3 (parameters: ‐t exon ‐g gene_id ‐s 0) to generate raw count matrices. Differential expression analysis was conducted separately for each dataset using DESeq2 v1.34.0, with biological replicates explicitly modeled in the design matrix. Genes with |log2(fold change)| ≥ 1 and Benjamini–Hochberg adjusted *p*‐value < 0.05 were classified as DEGs.

### 2.2 Cross‐Study Normalization and DEG Identification

To address technical variability across studies, we implemented a Random Forest‐based normalization approach using the randomForest R package (v4.7‐1.1). First, raw count matrices were variance‐stabilized transformed. A Random Forest classifier with 500 trees and mtry parameter set to the square root of features was trained to predict study origin. The out‐of‐bag residuals were then extracted to serve as batch‐corrected expression values, effectively removing study‐specific technical artifacts while preserving biological variation. Differential expression analysis was subsequently performed on these normalized data using DESeq2 v1.34.0, with biological replicates explicitly modeled in the design matrix. Genes exhibiting absolute log2 fold change ≥ 1 and Benjamini–Hochberg *p*_adj_ < 0.05 were classified as DEGs. Control conditions were standardized by verifying identical genetic backgrounds between control and stress‐treated samples within each study, confirming equivalent growth conditions before stress application, and cross‐referencing control sample metadata against original publications.

### 2.3 Identification of Shared DEGs

The DEG sets for drought, salinity, heat, and cold stresses were consolidated per stress type. To identify stress‐overlapping genes, we employed Jvenn for rigorous comparison of DEGs across the four abiotic stresses. Shared DEGs were defined as those simultaneously present in the DEG sets of all four stress types at stringent intersection criteria requiring detection in ≥ 80% of studies per stress category. Separate Venn diagrams were generated to visualize both upregulated and downregulated gene overlaps, enabling identification of conserved transcriptional responses across multiple abiotic stresses.

### 2.4 Expression Patterns and Transcription Factor Analysis of DEGs

To assess expression dynamics, the expression matrix of the 3237 shared DEGs was extracted and visualized as heatmaps using the R packages pheatmap (v1.0.12) and ggplot2 (v3.4.4). Gene IDs of DEGs were then submitted to the BMKCloud platform (https://international.biocloud.net) to predict potential TFs. The gene IDs of different TFs were linked to the gene expression matrices of these DEGs, and the gene expression patterns of these TFs under abiotic stresses such as drought, heat, cold, and salinity were depicted, respectively.

### 2.5 Weighted Gene Co‐Expression Network Analysis (WGCNA)

Co‐expression analysis was performed using WGCNA v1.72.1 in R (Langfelder and Horvath 2008). Soft thresholding power was set to 12 on the basis of the scale‐free topology fit criterion (*R* ^2^ > 0.85), as determined by the pickSoftThreshold function. Module detection employed hierarchical clustering with dynamic tree cutting using parameters minModuleSize = 30, deepSplit = 2, and mergeCutHeight = 0.25. This identified seven co‐expression modules under a correlation threshold of 0.85 for weighted associations. Network centrality metrics, including degree centrality (direct connections per node), betweenness centrality (fraction of shortest paths passing through a node), and eigenvector centrality (influence on the basis of neighbors' importance) were calculated using NetworkX v3.2. Hub genes were defined as the top 1% of nodes by intramodular connectivity (kWithin) with module membership correlation ≥ 0.9 to the module eigengene. Final network visualization was implemented in Cytoscape v3.7.2 using Group Attributes Layout.

### 2.6 GO and KEGG Enrichment of Shared Gene

The Eggnog mapper v2 database was employed to convert protein sequences from the reference genome for functional annotation and domain prediction. The resulting data were then employed to generate the OrgDB database using the Annotation Forge package (v1.36.0) in R software. GO annotation and visualization were conducted with the clusterProfiler (v4.6.2) tool. Significantly enriched terms were defined using a Benjamini‐Hochberg‐adjusted *p*‐value < 0.05, and enrichment maps were generated with the enrichplot and ggplot2 packages.

### 2.7 Abiotic Stress Experiment

Chinese Spring (CS) wheat ( *Triticum aestivum* L., AABBDD, 2n = 6 × 42) was used as the experimental material. High‐quality seeds were selected and surface‐sterilized with 1% sodium hypochlorite. The seeds were then placed on sterile, moist filter paper in disinfected petri dishes and kept in the dark for 48 h at room temperature. After 15 days, seedlings were grown under a 16 h light and 8 h dark cycle at 25/20°C with 70% humidity levels until reaching the two‐leaf stage. Subsequently, 10‐day‐old seedlings were treated with either 0 mM (control) or 150 mM NaCl for 24 h (Qin et al. 2020). During this period, the control group was kept in Hoagland solution at half strength, replaced every 2 days to remove metabolic byproducts potentially impacting growth (Liu et al. 2015). The heat stress protocol, on the basis of Tomas et al. (2022) with minor adjustments, involved keeping the control plants under normal greenhouse conditions, whereas the treatment group experienced 40/26°C (day/night) for 24 h. For cold stress, once the seedlings reached the two‐leaf stage, they were transferred to a separate environment at 4°C, whereas the control was maintained at 22°C (Li, Han, et al. 2023). Each treatment included three biological replicates, with three plants per replicate, totaling nine plants per condition. Leaves were harvested for RT‐qPCR analysis, whereas a second batch of plants under the same treatment was used for morphological and physiological measurements. Root systems were gently washed and photographed on black cloth (Sony camera, Japan).

### 2.8 Determination of Wheat Morphology and Chlorophyll Content

Following each stress treatment, clear morphological differences were observed between the control and treated groups. After carefully laying the wheat plants flat, plant height was measured from the base to the apex of the tallest leaf, and root length was measured from the base to the apex of the root. The lower portion of each wheat plant was then excised and placed in a 2 mL centrifuge tube. Fresh weight of shoots was measured on an analytical balance, and the tubes were covered with aluminum foil. According to previous methods for measuring chlorophyll content (Cheng et al. 2025), specifically, 1 mL of 80% acetone was added to each tube, and the samples were kept at 4°C in the dark overnight. The absorbance of the extracted solution was measured at 645, 663, and 652 nm. For dry weight measurements, samples were dried at 105°C for 30 min, then at 75°C for approximately 14 days until they reached a stable weight.

### 2.9 RNA Isolation and Quantitative Real‐Time PCR

To minimize batch effects, all samples were processed in randomized order, cDNA synthesis utilized identical master mixes, and interplate calibrators were included. Total RNA was extracted from harvested samples using TRIzol reagent, followed by DNase I treatment to remove genomic DNA contamination. Complementary DNA (cDNA) synthesis was performed using the PrimeScript RT reagent Kit (Takara) with oligo(dT) primers according to the manufacturer's instructions. Quantitative real‐time PCR reactions were performed with gene‐specific primers (Table S1 and Text S1). The stability of the wheat actin (*TaACT1*) reference gene was validated. Gene expression levels were calculated using the $2^{-∆∆C_{t}}$ method with three technical replicates per biological sample.

### 2.10 Statistical Analysis

Data were analyzed using one‐way analysis of variance (ANOVA) to determine statistical significance (*p* < 0.05). The three replications were set up in all experiments, and results are expressed as the mean ± standard deviation.

## 3 Results

### 3.1 DEGs Identification in Each Abiotic Stress

**FIGURE 1:**
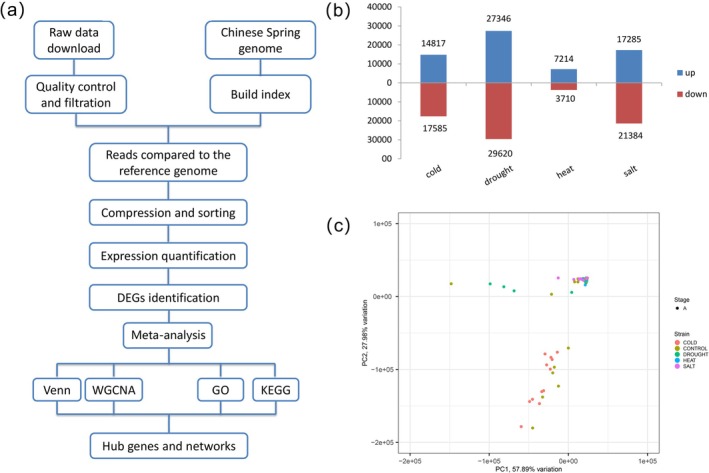
Bioinformatics analysis in DEGs identification. (a) The analysis process of hub genes in this study; (b) upregulation and downregulation of DEG number under cold, drought, high temperature, or salt stress; (c) principal component analysis (PCA) of all samples in transcriptome data.

Transcriptomic analysis of 100 samples from 10 independent studies revealed distinct expression profiles across four abiotic stresses (Figure S1 and Table S2). Analysis of 32 drought‐stressed samples identified 56,966 DEGs, comprising 27,346 upregulated and 29,620 downregulated genes filtered by the threshold of |log2FC| ≥ 1 and adjusted *p*‐value < 0.05. Examination of 15 heat‐stressed samples yielded 10,924 DEGs, including 7214 upregulated and 3710 downregulated genes. Evaluation of 28 cold‐stressed samples produced 32,402 DEGs, containing 14,817 upregulated and 17,585 downregulated genes, whereas analysis of 25 salinity‐stressed samples resulted in 38,669 DEGs, including 17,285 upregulated and 21,384 downregulated genes (Figure 1a,b). Principal component analysis revealed partial clustering of stress groups, with PC1 explaining 57.89% and PC2 explaining 27.98% of variance. The observed overlap in transcriptional profiles indicates shared molecular response mechanisms across multiple abiotic stresses (Figure 1c).

### 3.2 Conserved Stress‐Responsive Genes and Functional Enrichment

**FIGURE 2:**
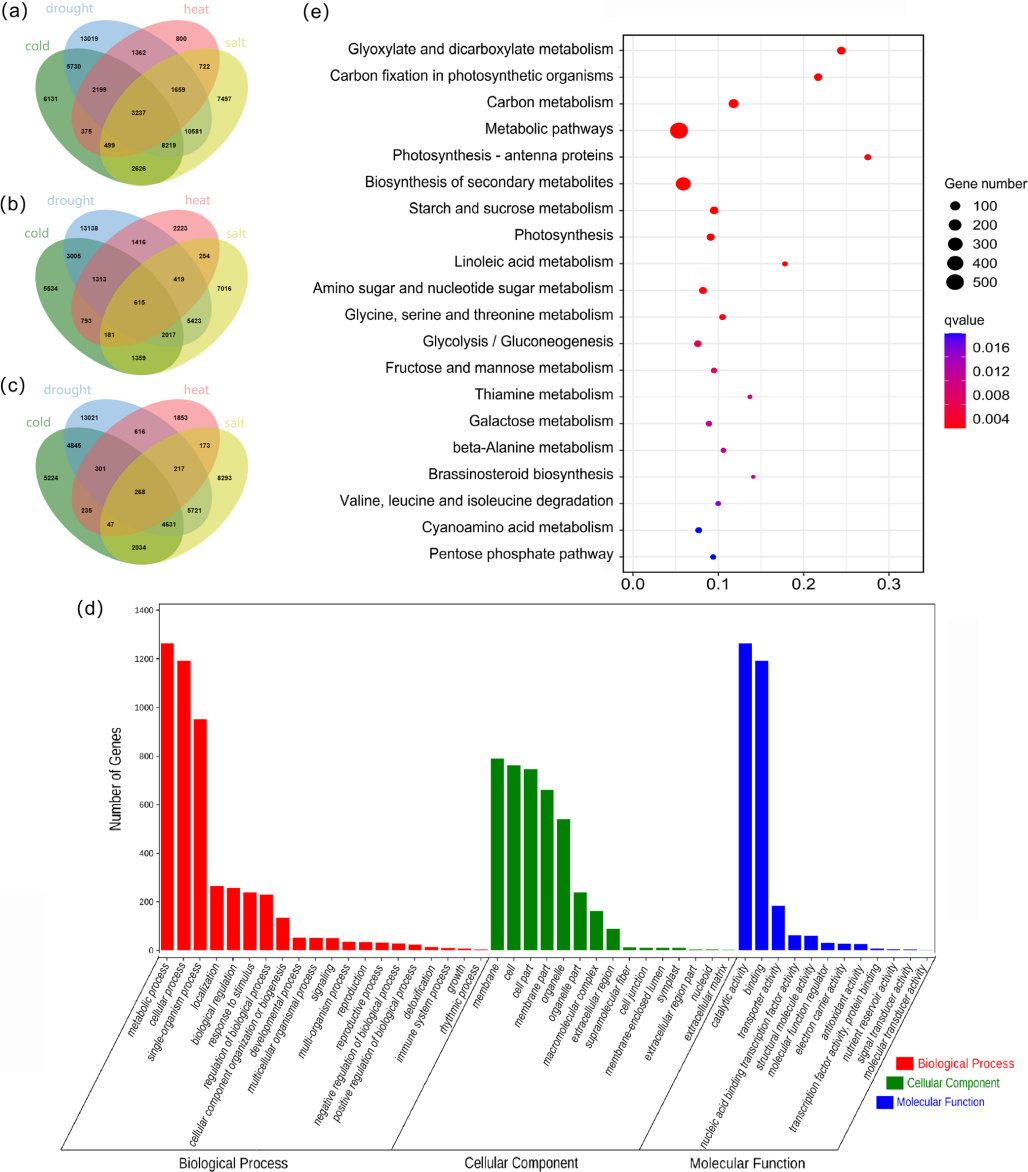
Venn diagram and annotation analysis of DEGs. (a) The Venn diagram of DEGs under cold (green), drought (blue), heat (pink), and salt stresses (yellow); (b) the Venn diagram of upregulated DEGs under cold, drought, heat, and salt stresses; (c) the Venn diagram of downregulated DEGs under cold, drought, high temperature, and salt stress; (d) GO annotation of 3237 shared genes, the red terms represent enrichment in ‘biological process,’ green represents enrichment in ‘cellular component,’ blue represents enrichment in ‘molecular function’; (e) KEGG enrichment of 3237 shared gene.

Intersection analysis identified 3237 genes responsive to all four stresses, representing a core set of conserved regulators. This included 13,019 genes under drought, 800 under heat, 6131 under cold, and 7497 under salinity conditions. Within the shared gene set, 615 genes (19.0%) were consistently upregulated and 268 genes (8.3%) downregulated across the four abiotic stresses (Figure 2a–c). Gene Ontology enrichment revealed significant cellular component terms, including cell part and organelle (Figure 2d). Molecular functions featured catalytic activity and nucleic acid binding TF activity (GO:0003824; GO:0003824). Biological processes encompass response to stimulus and biological regulation (GO:0009987; GO:0044710). Critically, significantly enriched terms included “transporter activity” (GO:0005215), “nucleic acid binding transcription factor activity” (GO:0001071), and “response to stimulus” (GO:0050896). KEGG analysis highlighted critical metabolic pathways including “glyoxylate and dicarboxylate metabolism” (ko00630), “carbon fixation in photosynthetic organisms” (ko00710), “carbon metabolism” (ko01200), and “metabolic pathways” (ko01100), indicating coordinated metabolic reprogramming during stress adaptation (Figure 2e).

### 3.3 Expression Patterns of Shared Genes Under Four Abiotic Stresses

**FIGURE 3:**
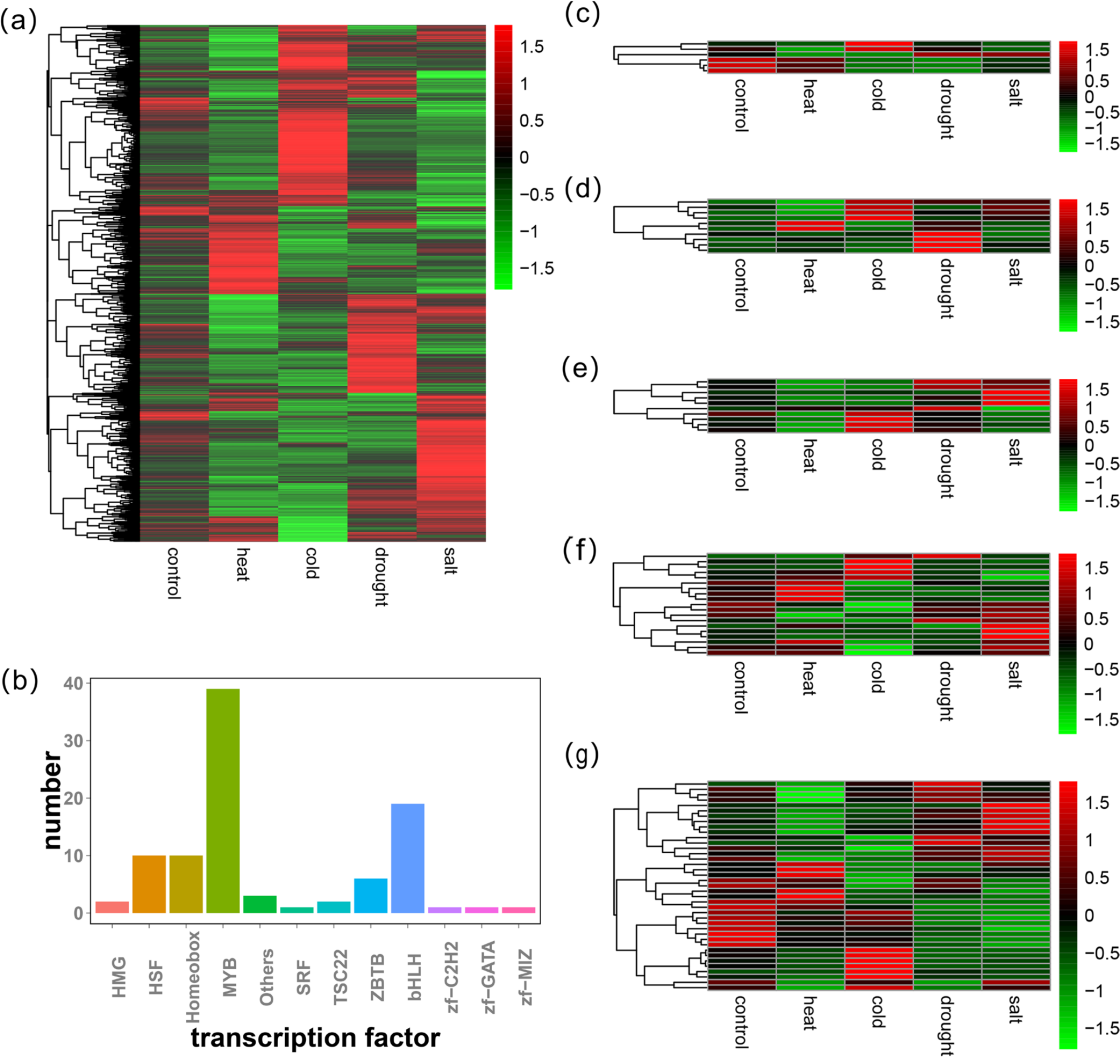
Analysis of transcription factors in 3237 shared genes. (a) Expression patterns of shared genes under four types of stress; red represents the highest level of expression, green represents the lowest level of expression; (b) the number and types of transcription factors in 3237 shared genes; (c) expression patterns of ZBTB type transcription factors under four types of stress; (d) expression patterns of HSF type transcription factors under four types of stress; (e) expression patterns of Homeobox type transcription factors under four types of stress; (f) expression patterns of bHLH type transcription factors under four types of stress; (g) expression patterns of MYB type transcription factors under four types of stress.

Expression profiling identified 53 universally upregulated genes encoding *protein phosphatase 2C* (*PP2C*), *phloem protein 2* (*PP2*), and *protein tyrosine kinase* (*TK*), alongside 219 consistently downregulated genes, including *glutamine synthetase* (*GS*), *inositol oxygenase* (*MIOX*), *glycosyl hydrolase 17 family* (*GH 17*), and *F‐box* (Figure 3a). Among 95 TFs identified from 3237 shared genes, 39 MYB factors were most abundant, whereas SRF, zf‐C2H2, zf‐GATA, and zf‐MIZ were minimally represented (Figure 3b). Moreover, the expression profiles of TFs under four different abiotic stress conditions were further examined. Among the members of the ZBTB TF family, two are upregulated under cold stress, one is upregulated under drought and salt stress, and the remaining three are all downregulated under the four abiotic stresses (Figure 3c). HSF TFs included two universally upregulated genes and one downregulated (Figure 3d). Homeobox TFs contained one universally downregulated gene (Figure 3e). bHLH factors exhibited three genes upregulated specifically under heat and cold, with one downregulated across the four abiotic stresses (Figure 3f). MYB TFs featured 1 universally upregulated gene and 11 downregulated (Figure 3g). These results indicate that there are regulatory factors that exert multiple regulatory effects under different abiotic stresses.

### 3.4 Identification of Hub Genes Responding to All Four Abiotic Stresses

**FIGURE 4:**
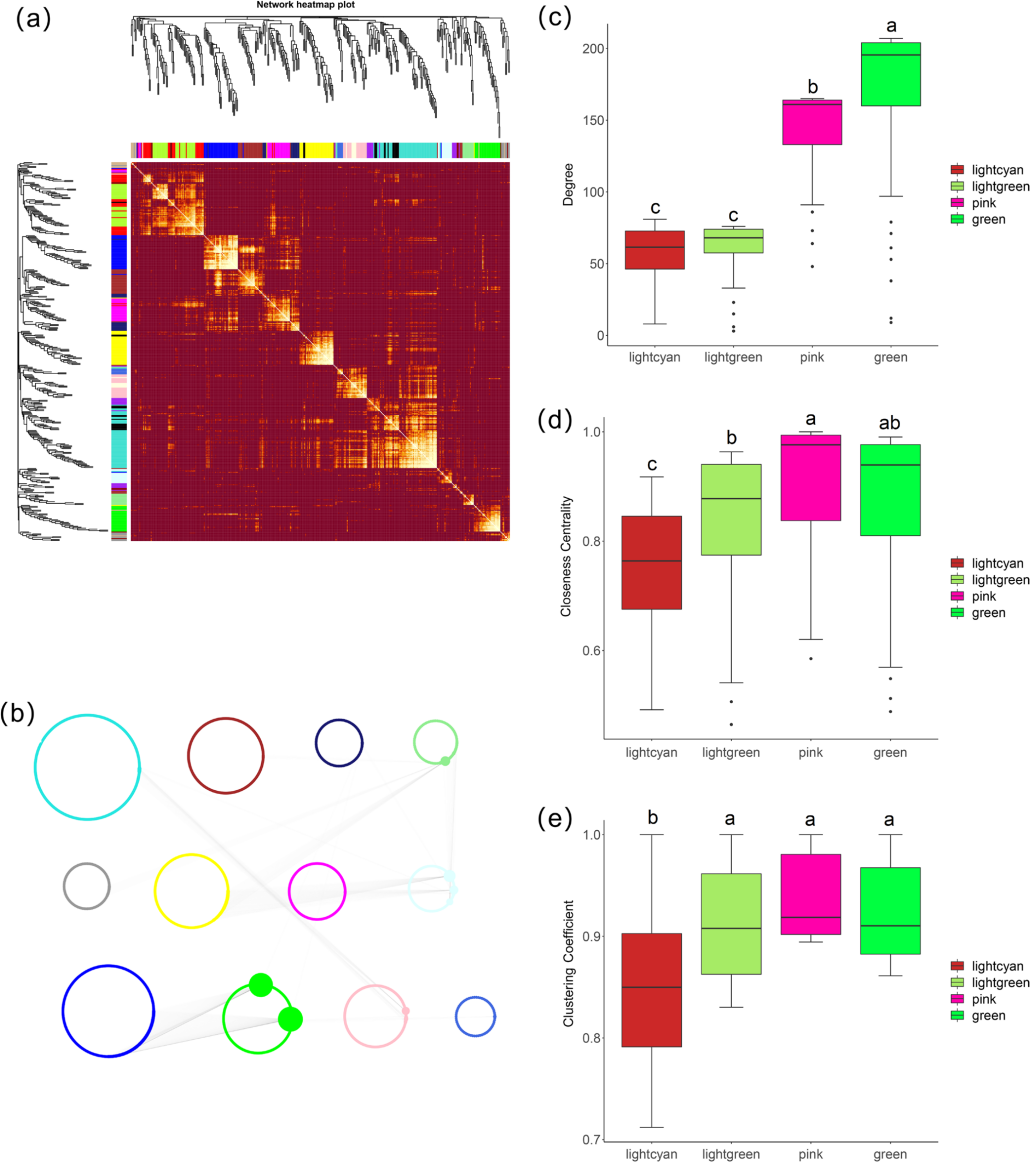
Weighted gene co‐expression network analysis (WGCNA) for hub genes' identification. (a) The network heat map of 3237 shared genes. Genes were clustered by dendrogram and topological overlap mapping, genes were clustered by dendrogram and topological overlap mapping. (b) A network of 3237 shared genes. The nodes with increased size are high connectivity annotations between different modules (with connectivity in the top 10%), and are identified as hub genes; (c) Average degree of each module; (d) Closeness centrality of each module; (e) The information centrality of whole co‐expression network.

Weighted gene co‐expression network analysis of 3237 shared genes identified 19 modules, with the largest module “turquoise” containing 479 genes and the smallest module “royal blue” containing 66 genes (Figure 4a,b). Eight hub genes exhibiting the highest intramodular connectivity (top 1%, module membership > 0.9, *p* < 0.01 for centrality metrics) were identified: one in light green, three in light cyan, two in green, and two in pink modules. Moreover, the average connectivity (Degree value) of green and pink modules was determined to be greater compared to that of the full co‐expression network (Figure 4c). The pink module displayed the highest centrality value (Closeness centrality) among the four modules, highlighting its critical role in response to the four abiotic stresses. The green module ranked second after the pink module and included two genes with high connectivity (Figure 4d). The average clustering coefficient of green, pink, and light green modules was higher than that of the light cyan module, suggesting that genes in those three modules have more similar expression patterns (Figure 4e). Functional annotation revealed hub genes encoding *Cotton fiber‐expressed protein*, *BES1/BZR1 plant transcription factor protein*, *Glycosyl hydrolase family 14*, and *RNA recognition motif*, showing module topology and hub gene positions.

### 3.5 GO and KEGG Analysis of Genes in Red and Brown Modules

**FIGURE 5:**
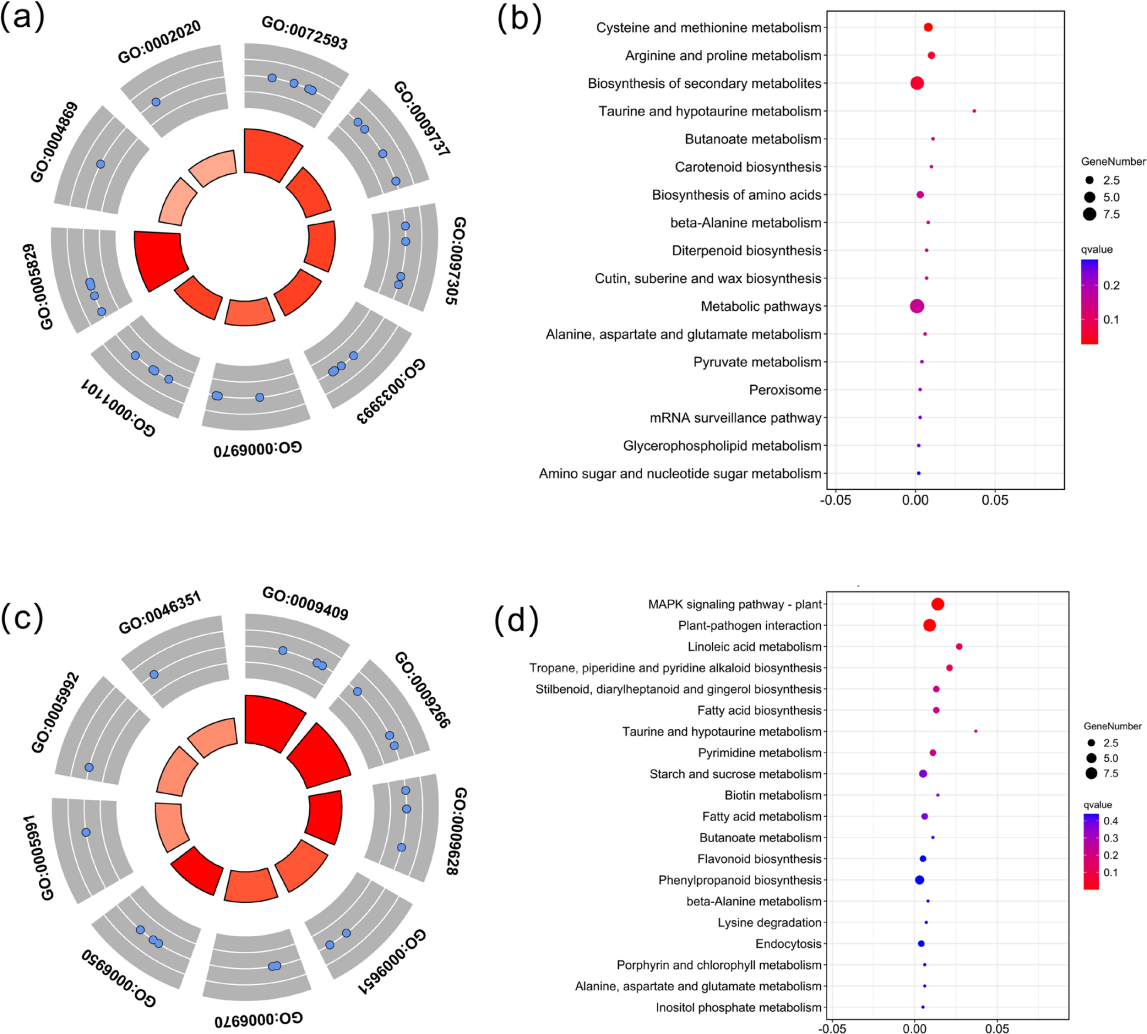
Annotation analysis of two modules (light green and green) highly correlated with stress. (a) GO annotation for light green modules; (b) KEGG enrichment analysis of light green modules; (c) GO annotation for green modules; (D) KEGG enrichment analysis of green modules.

In WGCNA analysis, eight hub genes were primarily concentrated in the green, light‐green, light‐cyan, and pink modules. To determine further function and regulation pathway, GO and KEGG analyses were conducted on those modules, especially the green and bright green modules, which contained the top node gene. Within the light‐green modules, nine GO pathways were identified (Figure 5a and Figure S3), including responses to ROS, metabolic process (GO:0072593), abscisic acid (GO:0009737), alcohol (GO:0097305), and cytosol (GO:0005829), among others. The KEGG enrichments contained “Cysteine and methionine metabolism (ko00270),” “Arginine and proline metabolism (ko00330),” and “Biosynthesis of secondary metabolites (ko01110)” (Figure 5b). In the green module, nine enriched GO terms included responses to cold (GO:0009409), temperature stimulus (GO:0009266), abiotic stimulus (GO:0009628), and stress (GO:0006950) (Figure 5c). KEGG annotations were significantly enriched in the “MAPK signaling pathway in plants (ko04016),” “Plant–pathogen interaction (ko04626),” and “Linoleic acid metabolic pathways (ko00591)” (Figure 5d). Most of the annotated pathways in these two modules are related to abiotic stress, indicating that the genes in these two modules are highly related to multiple abiotic stresses.

### 3.6 Changes in Plant Phenotype and Chlorophyll Content

**FIGURE 6:**
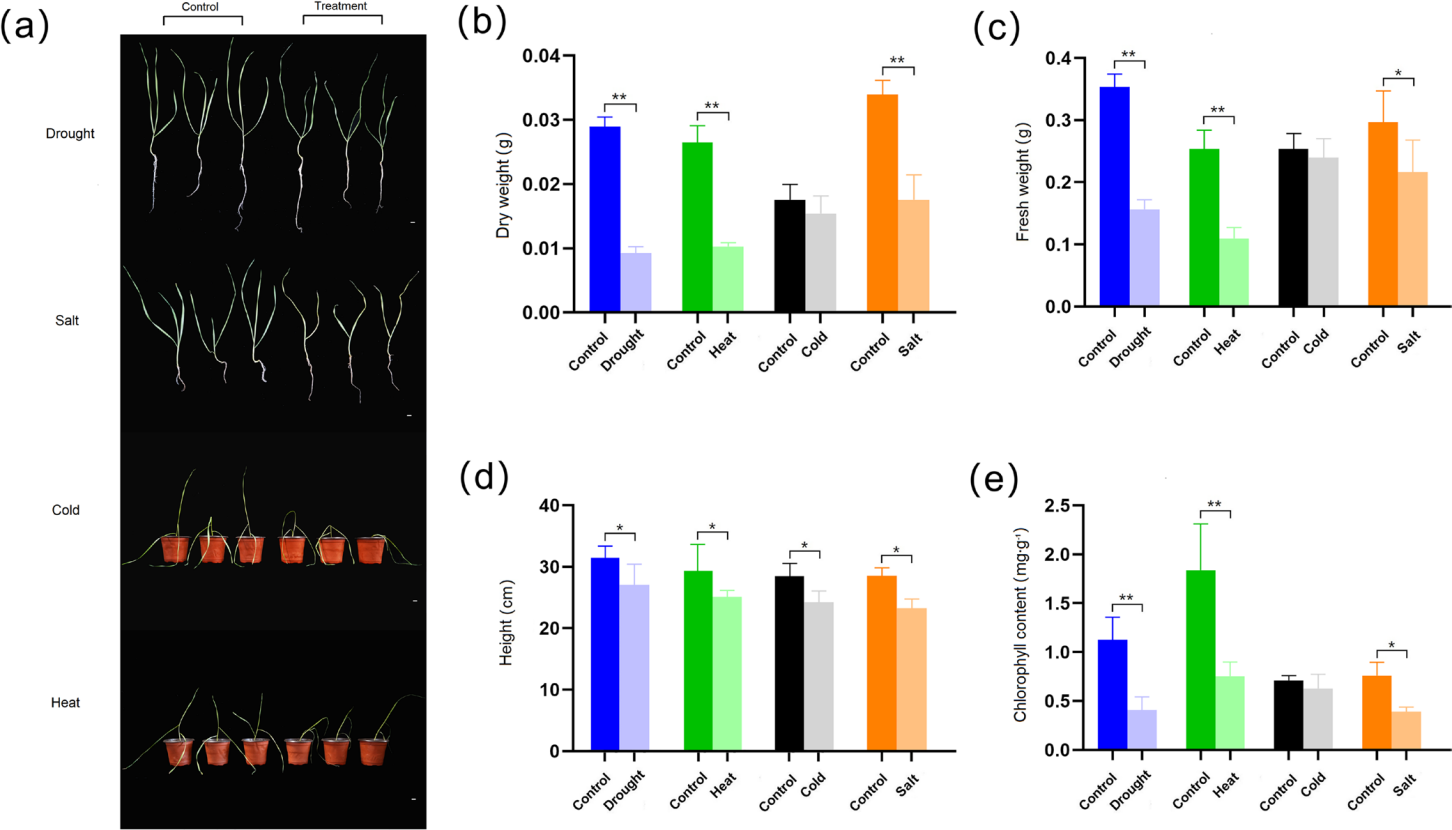
Evaluation of physiological and biochemical indicators of wheat under drought, heat, cold, and salt stress. (a) Wheat under 7 days of drought stress, 7 days of salt treatment, 7 days of cold treatment, and 1 day of heat treatment, respectively. (b) The significance of dry weight in wheat control group and treatment group under four types of stress; the value represented by each individual column in the bar chart is the average of three biological replicates. (c) The significance of fresh weight of wheat control group and treatment group under four types of stress. (d) The significance of plant height in wheat control group and treatment group under four types of stress. (e) Significance of chlorophyll content in wheat control group and treatment group under four types of stress; significance was calculated by one‐way ANOVA in SPSS16. *p ≤ 0.05; **p ≤ 0.01.

Stress treatments induced significant morphological alterations, manifested as slow growth, abnormal water loss, and color changes in the four abiotic stresses (Figure 6a). The differential analysis was performed on plant height, dry and fresh weight, and chlorophyll content to visualize the differences in wheat between the control and treatment groups. Dry and fresh weight showed significant differences between the control and treatment groups under the other three types of stress, except for cold stress. The dry weight was significantly decreased by 68.09%, 61.21%, and 48.33%, and the fresh weight was decreased by 55.66%, 56.58%, and 26.97%, after exposure to drought, heat, and salt stress, respectively (Figure 6b,c). Moreover, the plant height was significantly decreased by 14.00%, 14.45%, 14.77%, and 18.48%, under drought, heat, cold, and salt stress, respectively (Figure 6d). The chlorophyll content had no significant difference under cold stress, but it was decreased by 63.7%, 59.09%, and 48.32% under drought, heat, and salt stress, respectively (Figure 6e). The results suggested that although different abiotic stresses elicit specific physiological responses, alterations in underlying physiological processes are common to multiple stresses.

### 3.7 Verification of RNA‐Seq Results by RT‐qPCR

**FIGURE 7:**
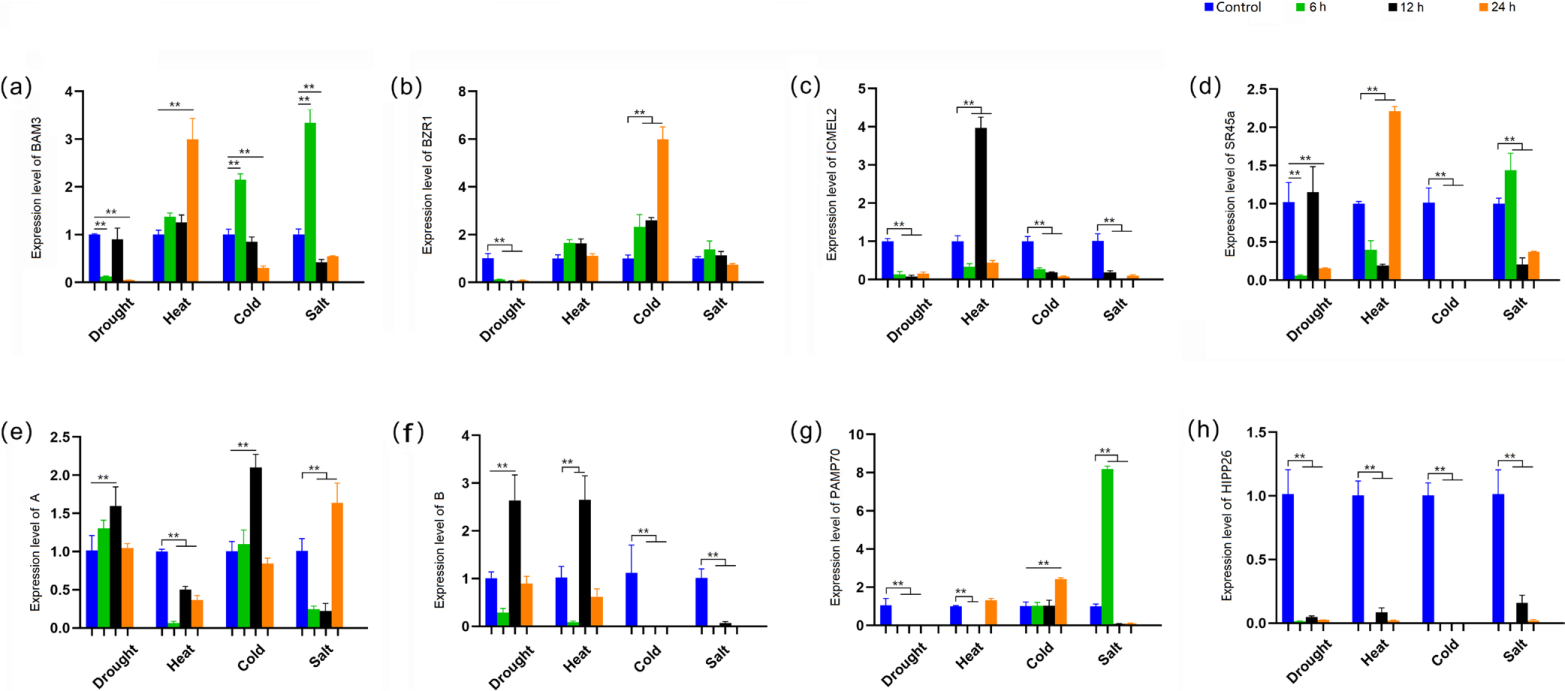
Differential expression of eight key genes under four different treatments. (a) Differential expression of BAM3 under four different stresses. (b) Differential expression of BZR1 under four different stresses. (c) Differential expression of ICMEL2 under four different stresses. (d) Differential expression of SR45a under four different stresses. (e) Differential expression of PAMP70 under four different stresses. (f) Differential expression of HIPP26 under four different stresses. The error bars show the standard deviation of the three replicates, the “**” indicates *p* ≤ 0.01.

Bioinformatics screening identified eight key genes predicted to be upregulated under drought, heat, cold, and salt stress, which were further confirmed by RT‐qPCR. Under drought stress, the expression level of *BAM3* was significantly upregulated by 1.99‐fold at 24 h of heat treatment, 1.14‐fold at 6 h of cold stress, and 2.33‐fold at 6 h of salt stress, compared to controls (Figure 7a). The expression level of *BZR1* was significantly increased by 1.99, 1.14, and 2.33‐fold of control at 6, 12, and 24 h of cold stress, but downregulated in drought stress (Figure 7b). *ICMEL2*'s expression was significantly increased by 2.94‐fold compared to the control at 12 h of heat stress, but was downregulated in drought, cold, and salt stress (Figure 7c). *SR45a* showed significant expression levels at 24 h under heat stress (1.21‐fold of the control) and at 6 h under salt stress (Figure 7d). *Gene A*, as an unidentified gene with unknown function, increased its expression levels by 1.58‐fold at 12 h of drought treatment, 2.09‐fold at 12 h of cold stress, and 1.63‐fold at 24 h of salt stress, whereas it was decreased under heat stress (Figure 7e). The expression level of *Gene B* showed significant increases at 12 h of drought treatment (1.62‐fold) and heat treatment (1.60‐fold), but it was downregulated in cold and salt stress (Figure 7f). *PAMP70*'s expression levels increased 1.39‐fold at 24 h of cold stress and 7.16‐fold at 6 h of salt stress (Figure 7g). *HIPP26* remained low expression level across the four abiotic stresses at all time points (Figure 7h). Quantitative PCR confirmed the association of these eight genes with multiple stresses, albeit to varying degrees. Gene regulation is complex, and factors within stress experiments or the simultaneous occurrence of multiple stresses in nature may prevent consistent upregulation at specific time points under all conditions.

## 4 Discussion

### 4.1 Analysis of Transcriptome Data Under Multiple Stresses

Adverse climate fluctuations have resulted in a reduction of arable land, extreme drought, abrupt temperature decreases, intense rainfall, and elevated soil salinity levels. Such abiotic stressors act as environmental limitations on crop growth and yield (Zhang et al. 2025). The significance of these findings is undeniable, especially concerning their potential value in genetic crop enhancement, but naturally occurring crops frequently experience multiple environmental challenges concurrently or at varying developmental stages, which can significantly reduce crop yields compared to singular stress (Mittler 2006; Prasad et al. 2011). To address the diverse socio‐economic and agricultural pressures brought on by climatic shifts, cultivating crops capable of enduring a wide range of abiotic stresses is crucial.

In this investigation, publicly available transcriptomic datasets were used, focusing on drought, heat, cold, and salinity stress in wheat, which led to the discovery of eight genes related to tolerance of multiple abiotic stresses. Specifically, the components responsible for withstanding various abiotic challenges were identified via bioinformatics reanalysis of 100 transcriptomes linked to four distinct stress conditions. A total of 3237 shared genes were identified across the four stress conditions. Despite applying strict selection criteria (FDR < 0.05, fold change > 2), some expression variation might also stem from dataset heterogeneity, which should be considered in downstream validation (Shojaee et al. 2022; Soltanpour et al. 2022). DEGs were rigorously screened for each stress individually, and the overlapping genes for the four types of stress were ultimately identified.

The analysis of DEG enrichment revealed various GO categories associated with stress responses, including notably enriched groups such as “biological regulation,” “metabolic processes,” and “cellular activities.” Importantly, the phrase “response to stimuli” was identified among the shared DEGs. Furthermore, the sub‐categories “response to stress” and “response to abiotic factors” were also identified among the shared DEGs. Terms such as “transporter function,” “regulation of molecular function,” and “antioxidant role,” which are linked to abiotic signaling pathways, were also annotated in this analysis.

For WGCNA, a scale‐free regulatory network was developed, consisting of 3237 common DEGs. The gene with the highest level of connectivity across different modules is designated as the hub gene. A total of eight hub genes were identified, with the GO functions of each module being annotated individually, revealing that four modules (Figure 4c–e) were associated with abiotic stress, leading to the selection of these hub genes as final central candidates. Gene BAM3 is mentioned in Kawaura et al. (2009). The article investigated the expression profiles of 3487 full‐length wheat cDNA genes, including this gene, in 28 tissues or treatments, indicating that these genes have a certain tolerance to abiotic stress, which is similar to our results. One gene corresponds to the BES1/BZR1 TF protein (Figure 4b), and two others belong to the Glycosyl hydrolase family 14. Although BES1/BZR1 has been studied in brassinosteroid signaling under single stresses in wheat (Yang et al. 2023), its consistent identification as a central hub across four concurrent abiotic stresses represents a novel finding in wheat meta‐analyses. Similarly, glycosyl hydrolase family 14 members have been implicated in stress responses (Da Ros et al. 2023; Roy et al. 2016), but their prominence as hub genes under combined stresses is less explored in wheat transcriptomics. In contrast, three hub genes identified in this study have no annotated function and have not been reported in previous wheat meta‐analyses, indicating they may represent novel stress‐responsive components (Fu et al. 2024; Garg et al. 2013). Preliminary domain analysis indicates the presence of no domain in plants. Orthologs in *Arabidopsis*' known abiotic tolerance‐related genes *AT3G03341* and *AT4G38060* suggested potential involvement in the stress response process. Further experimental validation of these unknown genes could uncover new regulatory mechanisms (Ibrahim et al. 2023; Wang et al. 2023). Instead of using extreme materials for validating tolerance genes against multiple stresses (Kumar et al. 2020), 100 transcripts were analyzed to gather information on a broader set of stress tolerance genes, and meta‐analysis was employed to identify shared genes. Additionally, a combination of GWAS and meta‐QTL analyses was applied to pinpoint genomic regions for multiple tolerance genes, providing a new perspective of considerable significance for exploring important multi‐resistance genes (Tanin et al. 2022).

TFs are essential in regulating responses to crop stress. A total of 95 TFs were identified among 3237 DEGs, including MYB, bHLH, HSF, and others (Figure 3b). Although many previously identified TFs such as MYB and HSF were again detected, the consistent presence of BES1/BZR1 as a hub gene across all four stress types is a novel observation in wheat studies (Da Ros et al. 2023; Gupta et al. 2019). This TF has previously been linked to brassinosteroid signaling under individual stresses, but its central regulatory role across multiple concurrent stresses may add new insight. This study explored both individual and combined stresses linked to heat, drought, and salt, with the coexistence of cold conditions being challenging alongside other stresses. BES1/BZR1 TFs were classified as hub genes. BES1/BZR1 is critical in plant growth, development, and adaptation to environmental stimuli. Regulation of brassinosteroid‐responsive genes was also attributed to BES1/BZR1 (Yu et al. 2018). Interestingly, the phenotypic results observed in this study—such as improved chlorophyll stability and reduced lipid peroxidation under stress—correspond well with the upregulation of BES1/BZR1 and glycosyl hydrolase genes, suggesting a strong link between molecular responses and physiological traits (Shah et al. 2018; Soltanpour et al. 2022). In Arabidopsis, BES1/BZR1 has been shown to regulate drought and cold tolerance through interaction with WRKY54, RD26, and other TFs, or by modulating the expression of CBF, WRKY6, PYL6, and RD26. Research indicates that genes conferring multiple abiotic stress tolerance are highly significant, especially when using transcriptomic data from public databases, with far‐reaching implications for crop improvement and resilience.

### 4.2 Tolerance Genes to Multiple Stress in Wheat

Few functional genes have been validated for tolerance to single or multiple abiotic stresses in wheat. In prior research, wheat plants with silenced *TaWRKY31* gene expression showed reduced water content under drought stress and also showed reduced expression of the *TaSOD*, *TaPOD*, *TaCAT*, and *TaPP2C* genes (Ge et al. 2024). *TaFKBP62*, which interacts with *TaBI‐1.1* and is localized in the endoplasmic reticulum, can confer heat stress tolerance (Lu et al. 2018). TaGRF6‐A, in association with the MYB TF TaMYB64, positively regulates salt stress tolerance (Shao et al. 2021). The *TaFER‐5B* gene, located on chromosome 5B, plays a significant role in enhancing tolerance to heat and other abiotic stresses and is involved in ROS scavenging. Overexpressing the AREB subfamily gene *TaABL1* within the bZIP TF family enhances wheat's response to ABA, induces stomatal closure under stress conditions, and improves tolerance to a range of abiotic stressors (Xu et al. 2014). Most genes in wheat that act on abiotic stress are TFs, with very few genes demonstrating the ability to simultaneously resist multiple abiotic stresses. Therefore, enhancing research efforts in this field, particularly for genes conferring multi‐stress tolerance like those identified here (BES1/BZR1, Glycosyl hydrolases, and novel hubs), is crucial.

Coordination of environmental stimuli and endogenous hormone signals is crucial for completing the life cycle. BES1‐EMS SUPPRESSOR 1 (BES1) and BRASSINAZOLE RESISTANT 1 (BZR1) are key components in the brassinosteroid (BR) signaling pathway (Wang et al. 2014). BES1 and BZR1 form a complex network with other protein factors, closely linked and jointly regulating the entire growth and development process of plants (Cao et al. 2024). Brassinosteroids are involved in plant responses to a variety of abiotic and biotic stresses, enhancing stress resistance in plants (Otani et al. 2022).

According to amino acid sequence alignment and hydrophobicity analysis, glycosyl hydrolases can be divided into multiple families. Obviously, each family member has a similar three‐dimensional structure. Glycohydrolytic enzymes have been classified into 111 families because of amino acid sequence similarity (Intra et al. 2008). Chitin is a type of β‐ The linear polymer of acetylglucosamine linked by 1,4 is recognized as the second most abundant polysaccharide in nature after cellulose (Wan et al. 2024). A chitin‐binding protein was found to be involved in drought response by inducing stomatal closure under abiotic stress in crops. These findings suggest that glycosyl hydrolase plays a significant role in crop abiotic stress responses.

### 4.3 The Significance of Multiple Resistance Genes in Production

Plants in natural environments do not always encounter optimal conditions. At times, essential factors for growth and development are lacking, which can lead to plant death. Ongoing shifts in environmental factors are a primary factor causing yield losses (Gupta et al. 2020). On cultivated land, short‐term drought or high temperatures might not significantly affect plant health and final yield. However, in nature, it is common for two or more types of pressure situations to coexist and may also have cumulative effects. The combination of two or three stresses can lead to a new stress state in plants, which can exceed the synergistic harmful interaction of individual stress effects (Balfagón et al. 2022; Zandalinas and Mittler 2022; Zandalinas et al. 2021). From this, it can be inferred that the damage caused by combined stresses to the nutritional and reproductive growth of plants can sometimes seriously threaten agricultural production. We used WGCNA to identify hub genes from 3237 common genes and made basic functional predictions of these hub genes through molecular biology, physiological, and biochemical analysis.

It is important to note key limitations of this study: (1) The reliance on in silico data from public repositories introduces potential confounding factors from heterogeneous experimental designs and platforms, despite normalization efforts (Shokri‐Gharelo et al. 2024; Tanin et al. 2022); (2) the variability in dataset sizes (e.g., fewer cold stress samples) may affect statistical power for certain comparisons (Da Ros et al. 2023; Fu et al. 2024; Gupta et al. 2019); and (3) the absence of functional validation for the identified hub genes remains a constraint, necessitating future transgenic or biochemical studies.

Overall, through the analysis of an open transcriptome dataset, we identified 3237 genes commonly expressed under four different abiotic stresses. Our findings reveal that the multiple stress‐tolerance genes we ultimately selected are linked to the BES1/BZR1 TF protein and glycosyl hydrolase family 14. We also identified three previously unknown genes. Subsequently, we subjected wheat to four types of stress treatments and measured various physiological and biochemical indicators. By integrating omics approaches and crop development, this study provides valuable new germplasm resources for future wheat breeding efforts.

## 5 Conclusions

This study identifies *BES1*/*BZR1* TFs and glycosyl hydrolase family 14 genes as central hubs in wheat under combined drought, heat, salt, and cold stress. They show consistent upregulation, high network connectivity, and functional links to chlorophyll preservation and oxidative stress mitigation. Three unannotated hub genes are flagged for validation. These molecular regulators provide actionable targets for breeding multi‐stress resilient wheat.

## Conflicts of Interest

The authors declare no conflicts of interest.

## Supporting information


**Data S1:** fsn370909‐sup‐0001‐supinfo.docx.

## Data Availability

All sequencing data obtained in this study are available in the NCBI database, and details can be found in Table S2.
