# Supplementary material for: Transcriptomic Meta‐Analysis Reveals Hub Genes Integrating Multiple Abiotic Stress Responses in Wheat
Source: Food Sci Nutr. 2025 Sep 8;13(9):e70909. doi: 10.1002/fsn3.70909 (PMC12415591; doi:10.1002/fsn3.70909)
Supplement: Supplementary file 1 — Data S1: fsn370909‐sup‐0001‐supinfo.docx. [file FSN3-13-e70909-s001.docx]

| **Primers** | **Primer (5'-3')** |
| --- | --- |
| BAM3_F | TACGACTGGGAGGGCTACGC |
| BAM3_R | CGCCGCACTGATGGAAGGAC |
| BZR1_F | CAAACACTGCGACAACAACG |
| BZR1_R | TGACACGGAGCACCCAATC |
| ICMEL2_F | TCAGTGCCACGGCGACCAA |
| ICMEL2_R | CTCTTCACCCTCTTCTTAGGC |
| SR45a_F | CGTGGATTTGGGTTTGTCA |
| SR45a_R | CGTCTTCGCTTTGCCTTCT |
| A_F | TGCCTTTCCGGGTCCGAGTC |
| A_R | TCCTGGTCACCGTCCGTCTG |
| B_F | CAGTGCGGGACTCGGAGCA |
| B_R | CTTGCGATGGCGGAACAGG |
| PAMP70_F | TCTCCTTCCCGTCCTCGTGC |
| PAMP70_R | CCGGCGTCCTCTGTTTCTCG |
| HIPP26_F | AGACGCTGGAGGGGATGGA |
| HIPP26_R | CGACGTAGCCGGTGACGGT |
| Actin_F | GACCGTATGAGCAAGGAGAT |
| Actin_R | CAATCGCTGGACCTGACTC |

**Table S1.** The primer sequence used in this study

| **Table S2.** The source and login number of transcriptome data in this study | | | | |
| --- | --- | --- | --- | --- |
| **Treatment** | **Title** | **Bioproject ID** | **Number of samples** | **Reference** |
| Drought | Temporal transcriptome profiling reveals expression partitioning of homeologous genes contributing to heat and drought acclimation in wheat (Triticum aestivum L.) | SRP045409 | 10 | Liu et al., 2015 |
|  | Transcriptome analysis of drought-responsive genes regulated by hydrogen sulfide in wheat (Triticum aestivum L.) leaves | SRP101470 | 4 | Li et al., 2017 |
|  | Transcriptomics Analyses Reveal Wheat Responses to Drought Stress during Reproductive Stages under Field Conditions | SRP102636 | 24 | Ma et al., 2017 |
| Heat | De Novo Transcriptome Analysis of Durum Wheat Flag Leaves Provides New Insights Into the Regulatory Response to Elevated CO2 and High Temperature | PRJEB34302 | 12 | Vicente et al., 2019 |
|  | Transcriptomic Analysis Reveal the Molecular Mechanisms of Wheat Higher-Temperature Seedling-Plant Resistance to Puccinia striiformis f. sp tritici | SRP107656 | 15 | Tao et al., 2018 |
| Cold | Comparative transcriptome profiling of a resistant vs susceptible bread wheat (Triticum aestivum L.) cultivar in response to water deficit and cold stress | PRJNA630059 | 9 | Konstantinov et al., 2021 |
| Salt | Transcriptome response of roots to salt stress in a salinity-tolerant bread wheat cultivar | SRP158842 | 4 | Amirbakhtiar et al., 2019 |
|  | Generation of new salt-tolerant wheat lines and transcriptomic exploration of the responsive genes to ethylene and salt stress | PRJNA549107 | 6 | Ma et al., 2021 |
|  | Comparative Analysis of the Glutathione S-Transferase Gene Family of Four Triticeae Species and Transcriptome Analysis of GST Genes in Common Wheat Responding to Salt Stress | PRJNA632706 | 12 | Hao et al., 2021 |
|  | Transcriptome analysis of bread wheat leaves in response to salt stress | SRP162799 | 4 | Amirbakhtiar et al., 2021 |


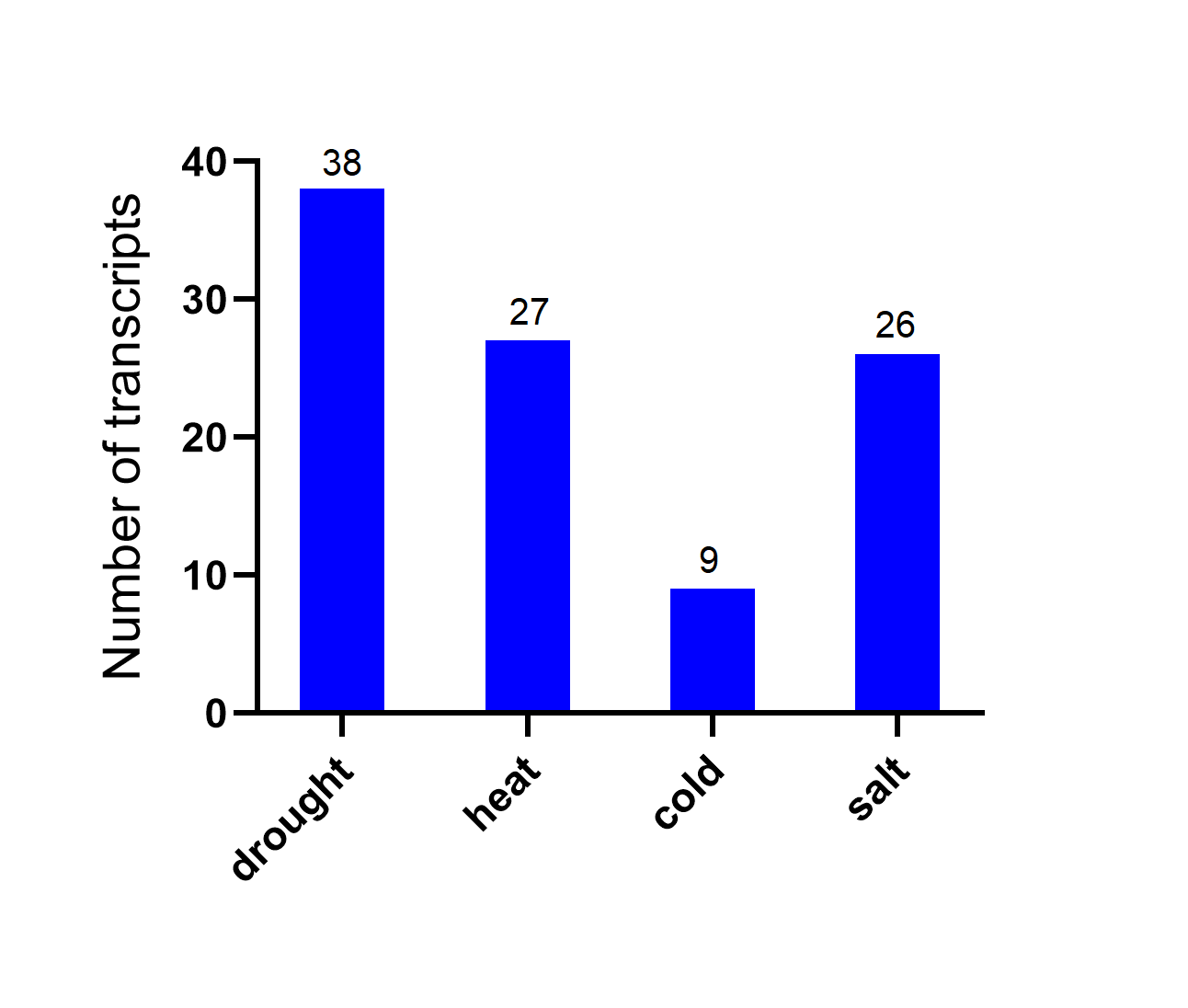


**Figure S1.** Number of samples in transcriptome data of drought, salt, heat and cold.


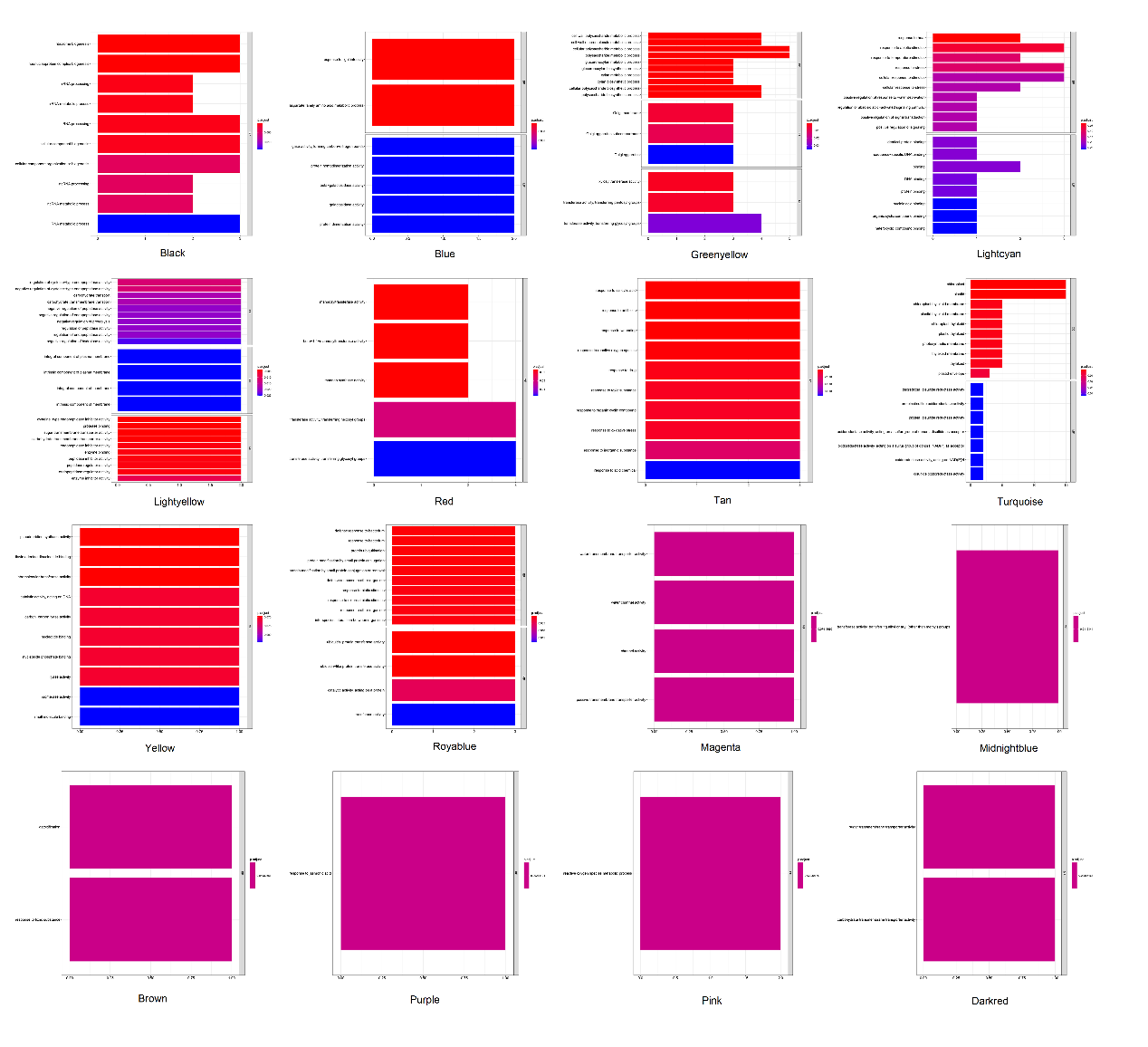


**Figure S2.** GO annotation information of genes in 16 modules. In the experiment, 3237 shared genes were GO annotated, and these genes were divided into 18 modules, and the green and light green modules that were more correlated with stress were selected, which were the GO annotation information of the remaining 16 modules.


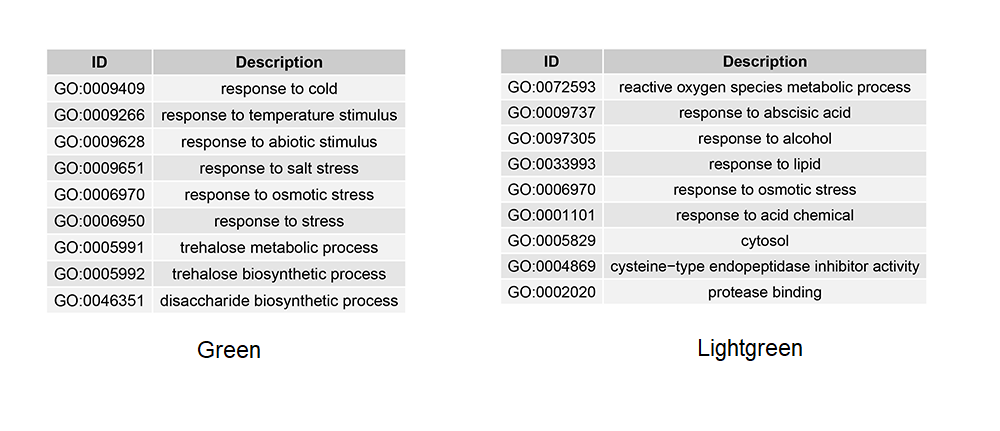


**Figure S3.** The description of the GO annotation in the green and light green modules.

**Text S1.** Sequence of 8 key genes

>TraesCS1B02G229000 (**BAM3**)

ATGGCGCTGACGCTGCGGTCCTCGACGTCCTTCCTCGCGCCGCTCGACCCCTGCTCCAAGCTCCTCCACAGGCCCGAGGACGCGCCGCCCTCCTGCGTCGCCGTCCCGCAGGCGCCCGCCAGGCTCCGCGCTCTCAGGGCGGCCGCGCAGGCCCCGCCGGCGCCGATGGAGACCCCGGCCCCGGCTCAGTCCGAGCTGCTGCACGGCCAGGTCCAGCAGGCGAACGCGGGGGGCGGCCAGCCGAGCAGGGGCGGGGTGCCGGTGTTCGTGATGCTGCCCCTTGACACGGTGGGGCCCGGCGGGCAGGTGTCGCGCGCGCGGGCGCTGGCGGTGAGTCTGATGGCGCTGCGGAGCGCCGGGGTGGAGGGGGTCATGGTGGACGTGTGGTGGGGCGTGGTGGAAAGGGATGGACCCGGACGGTACGACTGGGAGGGCTACGCCGAGCTGGTGCGAATGGTGGAGCGCGCCGGCCTCCGCCTCCAGATGGTCATGTCCTTCCATCAGTGCGGCGGCAACGTCGGCGACTCCTGCAACATCCCTTTGCCGCCATGGGTTCTGGAGGAGGTGAACGCCGACACGGACATCGTGTACACGGACAGGTCCGGCCGCCGCAACCCCGAGTACATCTCCCTCGGCTGCGACACGCTGCCGGTGCTCAAGGGCCGGACCCCCGTCCAGGTCTACTCCGACTACATGCGCAGCTTCCGCGACAGGTTCAGCGGCTACCTTGGCACCGTCATCGCCGAGGTCCAGGTTGGCCTGGGCCCCTGCGGCGAGCTGAGGTATCCTTCCTACCCGGAGGCCAATGGGACATGGAGTTTCCCGGGCATCGGCGAGTTCCAGTGCTACGACAAGTATATGCGGGCGTCGCTGCAGGCGGCGGCCGTGGCGGCGGGGCACGAGAACTGGGGGACAAGTGGGCCGCACGACGCCGGTGAGTACAAGCAGTTCCCGGAGGAGACGGGCTTCTTCCGGCGGGACGGCACGTGGAGCACCGAGTACGGCCACTTCTTCCTCAAGTGGTACTCCAGGATGCTCCTGGAGCACGGCGACCGCGTGCTGGCCGCTGCCGAGGCCATCTTCGGCGGCACTGGCGTGACGCTGTCTGCCAAGGTCGCCGGCATCCACTGGCACTACCGGACCCGCTCCCACGCCGCCGAGCTCACCGCGGGGTACTACAACACGCGGCATCACGACGGGTACGAGCCCATCGCGCAGATGCTGGCCAGGCACGGCACCGTGCTCAATTTCACGTGCATGGAGATGAAGGACGAGCAGCAGCCGGGCCACGCCGGCTGCTCGCCGGAGCTCCTGGTGCAGCAGGTCAGGGCCGCGGCGCGGGCGGCGCGCGTGGAGCTCGCCGGCGAGAACGCGCTGGAGCGGTACGACGAGCAGGCGTTCGCGCAGGTGGCCGCCACCGCCGAGGCCGCCGGGCTGAGCGCGTTCACGTACCTGCGCATGAACCGGAACCTGTTCGACGGCGACAACTGGCGACGGTTCGTCGCGTTCGTGAAGACCATGGCCGACGGCGGCGCGAGGACGGCGCTGCCCAGGTGCGACACTGGGCACTCGGATCTGTACGTGGGGTTCGTTGACGCCGCCAAGGAGCAGAGGGCGCCAGAGTCCGAGGCCGCCGCGGCATTGTAG

>TraesCS3A02G139000 (**BZR1**)

ATGGCGACGGGGGGAGGCGGAGGAGGAGGAGCGGCGGACTTCGGGGCGGCGGGGGGAGCGGGCGGCAGGATGCCGACGTGGAGGGAGAGGGAGAACAACAAGCGGAGGGAGCGGCGGCGGCGCGCGATCGCCGCCAAGATATTCTCCGGCCTGCGGGCGCACGGCGGGTACAAGCTGCCCAAACACTGCGACAACAACGAGGTCCTCAAGGCCCTCTGCAACGAGGCCGGCTGGGTCGTCGAGCCCGACGGCACCACCTACCGCAAGGGATGCAGACCTGCAGAACGCATGGATGGGATTGGGTGCTCCGTGTCACCAAGCCCATGTTCCTCATATCAGCCAAGTCCGCGGGCATCATACAATGCGAGCCCTACCTCCTCTTCATTCCCCAGCGGCGCGTCTTCGCCCTTCCTCCCGCATTCTAACAACATGGTTAATGGCGTTGATGCAACCCCCATCCTACCTTGGCTGCAAACGTTCTCCAATTCGACGGCATCGAATAAGCGGCCGCATCTTCCGCCGCTGCTGATTCACGGGGGCTCCATTAGCGCCCCGGTGACTCCTCCACTGAGCTCACCGACTGCTCGCACCCCTCGCATGAAGACAGACTGGGACGAGTCGGTGATCCAGCCTCCGTGGCATGGTTCAAACAGCCCTTGCGTGGTGAACTCCACCCCGCCAAGCCCCGGGCGTCAAATGGTTCCTGACCCGGCATGGCTGGCCGGCATCCAGATCTCGTCAACGAGCCCTTCATCGCCCACATTCAGTCTCATGTCCTCCAACCCATTCAGCGTCTTCAAAGAAGCCATCCCTGGCGGCGGTTCATCAAGGATGTGCACGCCGGGGCAGAGCGGCACGTGCTCGCCGGTGATCCCTGGCATGGCGCGGCACCCGGACGTTCACATGATGGACGTGGTCTCCGACGAGTTCGCGTTTGGAAGCAGCACCAACGGCGGCGCTCAGCAGGCCACCGCCGGACTGGTGAGGGCGTGGGAGGGCGAGAGGATCCACGAGGACTCCGGATCGGACGAGCTGGAGCTCACTCTCGGGAGCTCCAGGACGAGGAGCTGA

>TraesCS3A02G231100 (**ICMEL2**)

ATGGCATCCCTGAAGGCCGTGAAGCCAACCGGACTTGAAGGGCAGGCCAAGGAGCCTACCAAGGTTAGTGCCGCCAAGGGGCCTGCGAAGCTCAGTGCCACGGCGACCAAGCCAGCAGCCGCCAAGGGTGGCATCAAGAAGGCCGAGTCAAAGCCACGGGAGCCTAAGAAGAGGGTGAAGAGCTCCAAGCCAGCAGCAGCAAAAAACTGA

>TraesCS4A02G091300 (**SR45a**)

ATGTCGTATTCGAGGTACAGGAGTCGTTCAAGGAGTGTGGACTCGAGTGATGTTGAGAACCCTGGGAACAATCTCTTCGTGACTGGTTTATCATCTCGTCTAACTGATCAAGATCTGGAGAAGCATTTCTCTACAGAGGGAGAGGTGATTGATGCAAGTATAGTACTTGATCCATGGACAAGGGAATCACGTGGATTTGGGTTTGTCACCATGGCTACTCTTAAGGAGGCAGAACGCTGCATCAAATATCTCGACCGTTCAGTGCTGGAAGGTCGTGTCATTACTGTTGAGAAGGCAAAGCGAAGACGAGGTCGAACCCCAACACCGGGAAGGTATCTGGGCGCCAAATCATCGCGTGGAAGGAGGTATTCCCGAAGCAGGTCACCTGTTAGGAGAGACCGTTACAGCTCACGCTACTCGCCTGAGCGAGAACGCTCTTATTCTCCTTATCGCAGAGGACGATCATACTCTCGTCGTGACAGGCGTAGATCATACTCCCGAAGGAGATCATACACTCCTTCCGACAGGTCGGAGTCTCCCTACGACAGGCGAAGGTCCTACTCACCGTACGACAGGCGCATATCTTACTCACCCCACCATGGTCACCGCCACCGTTCAAGATCTCCATACCGTTACAGTAGGCGGAGGTCGCGCTCCCACGACCGTTCTGTTTCAGCATACTACAGCAGGCGCTATTCTCCAAGGAGCAGAAGACGGAGCTACTCTCGCAGCATATCGCCACGCAGGAGCTACTCCCGCAGCTGCTCCCCGGCGTCAGAAGAATCAAGGAGCTGTTCTCCGCGGAAAGGACGCGCCGGAAGCAAGGCATCGCGCAGCAGGTCACCTGGGAAGAGGCGTTCCAGAGAAAGCTATGCTCACAGCCGCAGTTCATGCTCGAGGTCCGTCTCCAGGGAGCGCTCAACCTCAGCAAGCACCTGA

>TraesCS5B02G196900 (**A**)

ATGGCCATGGGGCTCGGCCGATTCACACACTGGCTCTGGCCGGGTAACGCCGCGCGAGTTGGCAACCACGAGCTCCCCGGCATCGCCTTGACGGGCGCCTCCTTCCCCGAGTTCCCCTCCGGGTTCCGTGAGGCGGACGCTATCGCCTTCTCCAGTGCCGCCGCCGGCCGCCGCACGCGGCCGAGGAGGGTCAAGAACCAGCGGCGCAGTCGCGGGGAGCCCAGAATTGACAGGGAGTACGACATGGTCATCGTGCCGTCCGACGGCGGCGGGTGCCTTTCCGGGTCCGAGTCCGACGACTCCGACTGGTCCATCGGCTGGCTGGAGCCGCAGGCGCCGGAGATGCAGACGGACGGTGACCAGGAGACCTCCTTCGCCGTCCTCGTGCCCTGCTATCGCCGCGGCCGCGCCGAGCAGCCTATGATGCCCCAGGGACGGTTCCTTGGCGCTGGCCCTCTCGCCGATGGCGGCCCCTCCGATGGAAAGAATTTTGTAGAACAGTGGCTTTCTTCCCTCCAGAACTGA

>TraesCS5D02G041400 (**B**)

ATGCCGTTCGTGCCGTCGTGCGTGCAGTGCGGGACTCGGAGCAACCCCTGCCGGTGCAAGGTGGTCGGGCCGACGCTGGGGTTCGTGGCCTTCGTGGTCACCGGCGTGGTTGAGTGGCCGCTGGGCGCGGCGGTGTACCTGTTCCGCCATCGCAAGGGCCGCCGCATCATGGGCCACCCCGCCAGGGTCGTCTACCCCCGCGTCACCAGAGCCATCCCCATCTAA

>TraesCS5D02G199200 (**PAMP70**)

ATGGCTGGAACGGAGTGGTGCTGGCCGCTGCCGGCGTGGATGGGCTCCGGCGCGGCGTGGTTCGTGGCCCTCAACCTCGTCGTGGGCGCCATCTTCGCCCTGTCGTCGCGTGCGCAGCCGCAGTCGCCCTCGCCGCGCCGCGCCGGGAGCGGGGGCGGGATCACGCGCAGGGCCTCGTCGGCGGTGCTGCAGCGCATCCGTTCCTTCAGCATCTTCTCCTTCCCGTCCTCGTGCTTCCACACCGCGGAGCCTAGCCCGGGCGCCACCGCCGCGGCCACCTTCCGAGAAACAGAGGACGCCGGGACGCCGACCAGGAGGTCGCCAGCTACACCGCGTGACCGTGTACCACGCCCACACGCGACGGCAGAGCCGGCGAAAGAGGACGCCGTGGAAGACGAGAACTCGATGAGCATGGACGAGGCGTACGCGCTCGCCCTGGCAGGGCGGCAGCGGGCGCCGCCGACGGAGGAGGAGGCGGCCGGGTCCGAGGTGGACGCCAAGGCGGAGGAGTTCATCCAGGGGTTCAAGGAGGACCTCAGGCAGCAGCGCCTCAACTCCATCTTCAACTACACCCAGATGCTCAAGCGCCGCGCCGCCGGCGGCCAGCCGCCTGCCGCACCAGAATGA

>TraesCS7A02G382700 (**HIPP26**)

ATGGGGTTCCTGGAGGCCCTGTCGGGGCTGTGCCGGTCGTGCCCGGCCCCCCTCACGCGCGGCCACCTGCAGAAGGGGCGGCAGCTGGAGACGGTGGAGATGAAGGTCCGGATCGACTGCGAGGGGTGCGTGAGCAAGATCCGCAAGACGCTGGAGGGGATGGACGGCGTCACCGGCGTCGACGTCGTCCCCAGGGAGAACAGGGTGACCGTCACCGGCTACGTCGACGCCGCCAAGGTGATGCGCCGCGTCGCGCGCAAGACCGGCAAGCGCGTCGAGCCGTGGCCCTACGTGCCCTACGACGTCGTCGCGCACCCCTACGCGCCCGGCGCCTACGACAAGAGGGCGCCCGCCGGGTACGTCCGCGACGTCATGGCCAACCCCGGCGGCGCGAACGCGTCCTTCGCGCGCGCCACCTCCACCGAGACTAGGTACACCGGGGCCTTCTCCGACGAGAACCCCAACGCGGCGTGCGCGATCATGTAG
